# Serologic Testing for Middle East Respiratory Syndrome Coronavirus and SARS-CoV-2 Cross-Reactive Immune Responses, the Netherlands

**DOI:** 10.3201/eid3211.260690

**Published:** 2026-11

**Authors:** Merel R. te Marvelde, Anna Z. Mykytyn, Matthijs P. Raadsen, Alexandre H.J. Bouffier, Sivana Baptista Varela, Rory D. de Vries, Corine H. GeurtsvanKessel, Bart L. Haagmans

**Affiliations:** Erasmus Medical Center, Rotterdam, the Netherlands

**Keywords:** MERS-CoV, viruses, respiratory infections, MERS, Middle East respiratory syndrome coronavirus, COVID-19, vaccination, cross-reactivity, severe acute respiratory syndrome coronavirus 2, SARS-CoV-2, serology, diagnostics, the Netherlands

## Abstract

Cross-reactive SARS-CoV-2–specific antibodies could compromise the specificity of MERS-CoV serology, potentially leading to misclassification of infection history. SARS-CoV-2 exposure induced MERS-CoV antibodies primarily targeting the conserved S2 domain, with occasional neutralizing capacity. Combining MERS-CoV S1 binding with neutralization, however, limited the influence of SARS-CoV-2–induced cross-reactivity on MERS-CoV serology.

Since its discovery in 2012, Middle East respiratory syndrome coronavirus (MERS-CoV) has caused small outbreaks in humans with case-fatality rates of ≈30%–40% (*1*,*2*). Primary cases typically result from direct contact with infected dromedary camels, but the virus can cause large outbreaks in healthcare settings (*2*). No licensed MERS-CoV vaccines are available, whereas COVID-19 vaccines were rapidly developed when SARS-CoV-2 emerged in 2019 (*3*,*4*). Serologic detection of MERS-CoV cases is essential for identifying previously undetected infections during outbreaks and surveillance among high-risk populations, providing insights into MERS-CoV epidemiology in settings where molecular testing is unavailable or incomplete (*5*,*6*). However, cross-reactivity induced by SARS-CoV-2 infection or vaccination could hamper detection of MERS-CoV–specific antibodies. 

The spike protein, the most immunogenic protein of coronaviruses, is used in mRNA- and vector-based vaccines and shares 58% similarity between SARS-CoV-2 and MERS-CoV (*7*). Several studies have demonstrated the presence of cross-binding antibodies after SARS-CoV-2 infection (*7*–*10*), sometimes with MERS-CoV neutralizing capacity (*11*–*13*). Such cross-reactivity can be difficult to distinguish from MERS-CoV–induced immune responses. World Health Organization (WHO) guidance for laboratory testing of MERS-CoV, published in 2018, states that a positive screening assay (e.g., ELISA) in combination with a positive result for neutralization indicates past MERS-CoV infection (https://iris.who.int/server/api/core/bitstreams/38aebc9e-4604-4640-9f7c-99e58d92d34a/content). No effect of SARS-CoV-2–induced cross-reactivity on those recommendations has been reported, but such effect would be critical for determining the specificity of MERS-CoV serology. We investigated the influence of different types of SARS-CoV-2 exposures on antibody cross-reactivity to MERS-CoV and consequences for MERS-CoV serology.

## The Study

We tested serum samples from persons in the Netherlands with different SARS-CoV-2 exposures (Appendix Table 1). Samples were obtained from patients with mild symptomatic COVID-19 infection (no hospital admission) and severe COVID-19 infection (admission to the intensive care unit), before COVID-19 vaccines were available. In addition, we included serum samples from persons who had received multiple COVID-19 vaccines; most had also experienced breakthrough COVID-19 infection (Appendix Table 2). The time of serum collection after SARS-CoV-2 exposure differed: 28 (interquartile range [IQR] 27–28) days after vaccination for the vaccinated group, 59 (IQR 41–69.75) days after symptom onset for the mild COVID-19 group, and 1 (IQR 0–1) day after intensive care unit admission for the severe COVID-19 group. Because the cohorts were established in the Netherlands, we assumed participants had not been exposed to MERS-CoV.

We measured SARS-CoV-2–binding IgG by in-house ELISA using full spike protein and the S1 and S2 subunits to determine SARS-CoV-2–specific immune responses in exposed persons. The pattern of binding antibodies was similar across groups for all antigens; serum from vaccinees with multiple SARS-CoV-2 exposures contained the highest levels of binding antibodies, followed by serum collected after severe and mild infection (Figure 1, panels A–C). The negative control prepandemic serum showed limited binding to SARS-CoV-2. To assess the functionality of the antibodies, we conducted a focus reduction neutralization test (FRNT) using SARS-CoV-2 with the spike mutation D614G. Neutralizing titers were again highest in vaccinated persons, followed by those with severe COVID-19 and mild COVID-19 (Figure 1, panel D).

**Figure 1:**
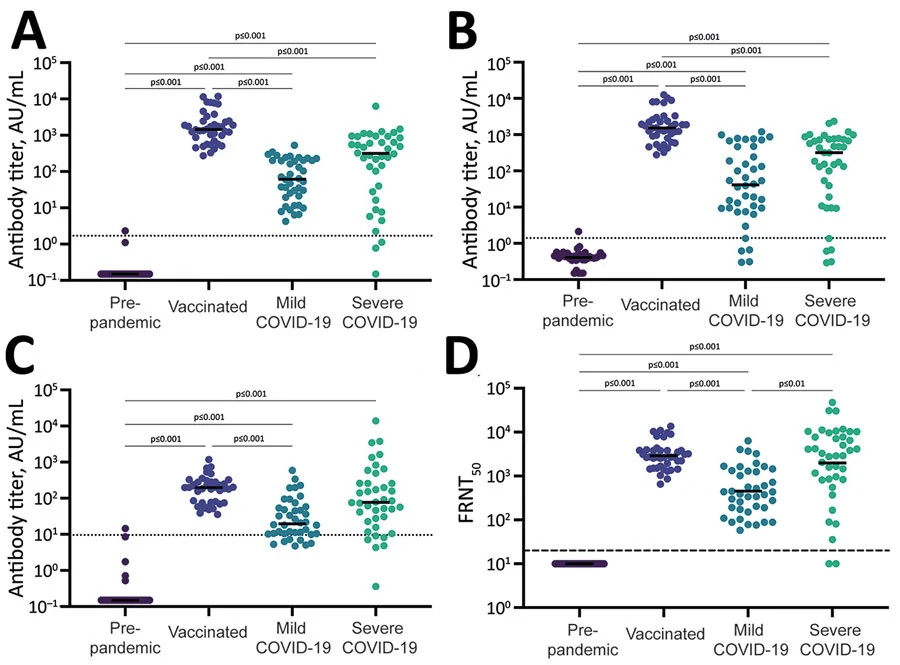
SARS-CoV-2 binding and neutralizing antibody titers after different SARS-CoV-2 exposures from study of serologic testing for Middle East respiratory syndrome coronavirus and SARS-CoV-2 cross-reactive immune responses, the Netherlands. Prepandemic serum samples were collected during 2015–2018, serum samples from persons vaccinated against COVID-19 were collected in 2023, and serum samples for persons with mild and severe COVID-19 were collected in 2020. A–C) Binding IgG to SARS-CoV-2 full spike (A), SARS-CoV-2 S1 (B), and SARS-CoV-2 S2 (C) were measured by in-house ELISA. Horizontal bars indicate medians; dotted lines indicate the cutoff for positivity on the basis of prepandemic samples. D) Neutralizing antibody titers were determined by focus reduction neutralization test by using SARS-CoV-2 D614G. Horizontal bars indicate geometric mean titer; dashed line indicates the lower limit of detection. We used a nonparametric Kruskal-Wallis test followed by a Dunn multiple comparisons test to evaluate the statistical significance between groups. FRNT_50_, 50% focus reduction neutralization test.

Next, we assessed cross-reactivity by evaluating MERS-CoV immune responses in the SARS-CoV-2–exposed groups. The antibody binding patterns for MERS-CoV were different than for SARS-CoV-2 (Figure 2, panels A–C). All SARS-CoV-2 exposed groups included serum that could bind MERS-CoV full spike, but binding was highest after severe COVID-19 (median 7.1 [IQR 1.2–78.3] IU/mL), followed by after vaccination (median 1.8 [IQR 0.7–4.2] IU/mL) and mild COVID-19 (median 1.1 [IQR 0.7–3.4] IU/mL). Cross-reactivity was mostly mediated by binding to MERS-CoV S2; MERS-CoV S1 binding was low but observed in several serum samples. The severe COVID-19 group had the highest MERS-CoV S2 binding antibody levels (median 67.0 [IQR 17.5–390.0] IU/mL) compared with the mild COVID-19 group (median 21.0 [IQR 9.6–36.9] IU/mL) and the vaccinated group (median 17.1 [IQR 7.9–30.1] IU/mL). The prepandemic group also showed some S2 binding (median 5.4 [IQR 3.7–11.2] IU/mL), possibly indicating cross-reactivity with other coronaviruses. We further assessed the MERS-CoV neutralizing capacity of the serum samples in accordance with the WHO recommendations for MERS-CoV serology. Of note, a subset of 11 patients from the severe COVID-19 group had detectable antibodies capable of MERS-CoV neutralization (Figure 2, panel D). One sample from the mild COVID-19 group partially neutralized MERS-CoV. Neutralizing antibody titers were 26–694.

**Figure 2:**
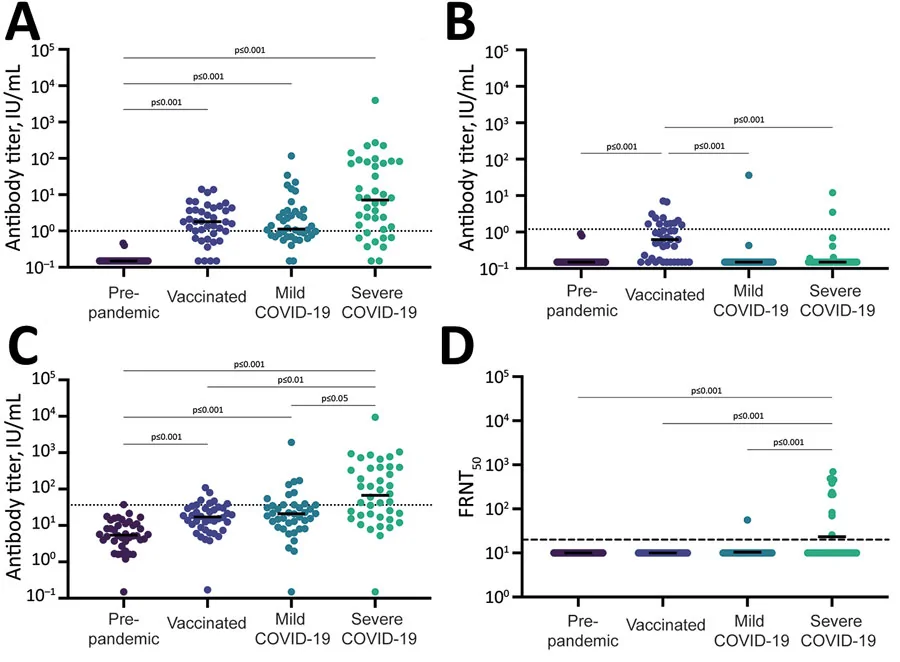
MERS-CoV binding and neutralizing antibodies after different SARS-CoV-2 exposures from study of serologic testing for Middle East respiratory syndrome coronavirus and SARS-CoV-2 cross-reactive immune responses, the Netherlands. Prepandemic serum samples were collected during 2015–2018, serum samples from persons vaccinated against COVID-19 were collected in 2023, and serum samples for persons with mild and severe COVID-19 were collected in 2020. A–C) Binding IgG to MERS-CoV full spike (A), MERS-CoV S1 (B), and MERS-CoV S2 (C) were measured by in-house ELISA. Horizontal bars indicate medians; dotted lines indicate the cutoff for positivity based on prepandemic samples. D) Neutralizing antibody titers were determined by focus reduction neutralization test. Horizontal bars indicate geometric mean titer; dashed line indicates the lower limit of detection. We used a nonparametric Kruskal-Wallis test followed by a Dunn multiple comparisons test to evaluate the statistical significance between groups. FRNT_50_, 50% focus reduction neutralization test.

To test the WHO-recommended 2-tier approach combining ELISA screening with a neutralization assay, we investigated the correlation between MERS-CoV neutralization and binding antibodies for all samples from persons who had severe COVID-19. MERS-CoV neutralizing antibody levels correlated with full spike binding (Figure 3, panel A); some serum samples were double-positive for binding and neutralization. Binding to full spike was most likely because of S2 binding (Figure 3, panel B). Those results indicate that MERS-CoV full spike–based and S2-based assays are likely to have limited specificity to detect past MERS-CoV infection because of cross-reactivity. However, MERS-CoV S1 binding was not correlated with neutralization (Figure 3, panel C) and only 1 sample from the SARS-CoV-2–exposed cohorts was double-positive, demonstrating that combining MERS-CoV S1 binding with neutralization minimizes the influence of SARS-CoV-2–induced cross-reactivity. SARS-CoV-2 binding and neutralization were not correlated with MERS-CoV neutralization (Appendix Figure 2), indicating that the level of the SARS-CoV-2 antibody response does not explain the potency of MERS-CoV neutralization.

**Figure 3:**
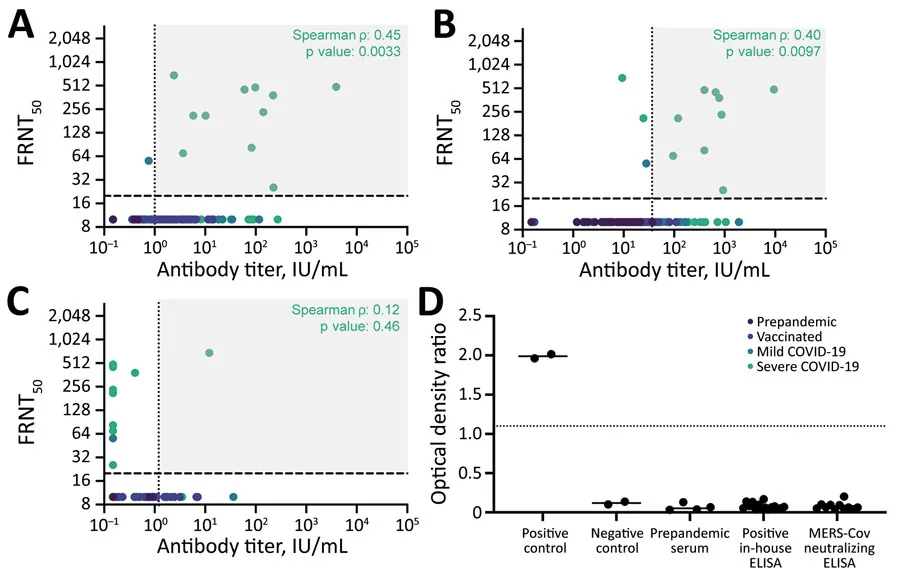
Correlation plot of MERS-CoV neutralization and MERS-CoV binding IgG levels from study of serologic testing for Middle East respiratory syndrome coronavirus and SARS-CoV-2 cross-reactive immune responses, the Netherlands. Prepandemic serum samples were collected during 2015–2018, serum samples from persons vaccinated against COVID-19 were collected in 2023, and serum samples for persons with mild and severe COVID-19 were collected in 2020. A–C) Serum samples from all groups were visualized showing the MERS-CoV neutralization titer and binding antibodies to MERS-CoV full spike (A), MERS-CoV S2 (B), and MERS-CoV S1 (C). For persons with severe COVID-19, the correlation between MERS-CoV neutralization and binding was determined by the Spearman rank-order correlation coefficient (ρ). Dashed horizontal lines indicate the lower limit of detection of the focus reduction neutralization test; vertical dotted lines indicate cutoff for positivity based on prepandemic samples for in-house ELISAs. Gray shading indicates double-positive samples capable of neutralizing MERS-CoV and binding to MERS-CoV spike in the in-house ELISA. D) A subset of prepandemic control serum samples (n = 4), serum samples positive on the in-house MERS-CoV S1 ELISA (n = 15), and MERS-CoV neutralizing serum samples (n = 11) tested on a commercial ELISA routinely used in MERS-CoV diagnostic testing according to manufacturer’s protocol. Assay controls provided by the manufacturer are shown. The optical density ratio depicts the optical density value of the sample divided by the calibrator. The dotted line indicates the recommended cutoff for positivity by the manufacturer (ratio >1.1). FRNT_50_, 50% focus reduction neutralization test.

Although MERS-CoV neutralization and MERS-CoV S1 binding were not correlated, several serum samples showed low-level binding to MERS-CoV S1. We tested serum samples that were positive by in-house ELISA and samples could neutralize MERS-CoV by commercial MERS-CoV S1 ELISA (Euroimmun, https://www.euroimmun.com). All samples were below the ratio for positivity (Figure 3, panel D), indicating that the commercial ELISA is less sensitive but more specific than the in-house ELISA to detect MERS-CoV–binding antibodies indicative of past MERS-CoV infection. Thus, the influence of SARS-CoV-2–induced cross-reactivity can be minimized by the combination of a MERS-CoV S1 ELISA with a neutralization assay or by using a commercial ELISA with a higher cutoff.

## Conclusions

We investigated antibody cross-reactivity between SARS-CoV-2 and MERS-CoV and effects on MERS-CoV serology. MERS-CoV–reactive antibodies after SARS-CoV-2 exposure are common in vaccinated and naturally infected persons (*7*–*10*), likely because the viruses have genetically similar spike proteins, mainly in S2 (*7*). Consequently, we found cross-reactivity primarily consists of binding to MERS-CoV full spike and S2, consistent with previous findings (*8*,*12*). The functionality of cross-reactive antibodies differed on the basis of SARS-CoV-2 exposure type. MERS-CoV neutralizing antibodies were detected in samples from persons with severe COVID-19 but not from vaccinated persons who were exposed to SARS-CoV-2 multiple times. Severe COVID-19 infection might expose the immune system to higher antigen loads and a different spike conformation than vaccination with prefusion stabilized spike, potentially promoting antibody responses to conserved S2 epitopes that might contribute to greater cross-reactivity with other coronaviruses (*14*).

The WHO MERS-CoV serology guidelines recommend confirming ELISA results with neutralization assays but do not specify antigens to be used or consider SARS-CoV-2–induced cross-reactivity. We found that combining MERS-CoV S1 ELISA with neutralization minimizes double-positive tests, decreasing the occurrence of false-positive results. Although we did find low levels of binding to MERS-CoV S1 using an in-house ELISA, we found no correlation between MERS-CoV neutralization and S1 binding. The samples tested negative in a commercial MERS-CoV S1 ELISA, suggesting that a high cutoff increases specificity but reduces sensitivity compared with the in-house ELISA. A higher cutoff could be applied to the MERS-CoV S1 binding assay, as in the commercial ELISA, but that approach reduces sensitivity, potentially failing to identify true MERS-CoV–positive cases with low S1 responses. The nucleocapsid protein is sometimes used in MERS-CoV serology (*15*), but SARS-CoV-2 infection can induce antibodies that bind to the MERS-CoV nucleocapsid, making that antigen less suitable for MERS-CoV serology (*9*). 

In conclusion, to minimize the influence of SARS-CoV-2–induced cross-reactive antibody responses, serologic testing for MERS-CoV should combine MERS-CoV S1 binding with a neutralization assay. That process would enable more accurate tracking of true MERS-CoV seroprevalence, enhancing surveillance and response efforts.

Appendix Additional information for serologic testing for Middle East respiratory syndrome coronavirus and SARS-CoV-2 cross-reactive immune responses, the Netherlands.
