# Supplementary material for: Serologic Testing for Middle East Respiratory Syndrome Coronavirus and SARS-CoV-2 Cross-Reactive Immune Responses, the Netherlands
Source: Emerg Infect Dis. 2026 Nov;32(11):1874–8. doi: 10.3201/eid3211.260690 (PMC13715889; doi:10.3201/eid3211.260690)
Supplement: Supplementary file 1 [file 26-0690-Techapp-s1.pdf]

Article DOI: https://doi.org/10.3201/eid3211.260690

EID cannot ensure accessibility for supplementary materials supplied by authors. Readers who have difficulty accessing supplementary content should contact the authors for assistance.

Serologic Testing for Middle East Respiratory Syndrome Coronavirus and SARS-CoV-2 Cross-Reactive Immune Responses

Appendix

Materials and methods

Cohorts

Sera from the four cohorts (pre-pandemic, vaccinated, mild COVID-19 and severe COVID-19) were obtained from participants enrolled in previously approved studies conducted at Erasmus MC. All original study protocols had received approval from the Erasmus MC Medical Ethics Review Committee, and participants had provided informed consent for the use of their study materials, including future research purposes. The XBB boosted cohort consisted of a subset of 40 randomly selected patients from the SWITCH-ON study who had received the XBB.1.5 booster vaccine (*1*). The mild COVID-19 cohort consisted of 40 randomly selected donors from the CONCOVID study obtained through Sanquin (*2*). The severe COVID-19 cohort included individuals admitted to the ICU due to SARS-CoV-2 infection whose samples were collected as part of the longitudinal CIUM biorepository study. Serum samples collected from healthy individuals before the COVID-19 pandemic, originating from the COVA study, were used as a control group (COVA study). The studies from which serum samples were obtained for the present analysis were approved by the Medical Ethics Review Committee of Erasmus MC; MEC-2022-0462 (SWITCH-ON), METC-2020-0682 (CONCOVID), MEC-2020-0222 (CIUM), MEC-2014-398 (COVA study). All sera were heat inactivated for 30 min at 56°C.

Viruses

SARS-CoV-2 isolate 614G/Bavpat-1 (Genbank: OM304632, D614G variant) was isolated from a oronasopharynx swab of a patient in April 2020 in the Netherlands. MERS-CoV/EMC isolate HCoV-EMC/2012 (Genbank; NC_019843.3) was isolated from sputum of a patient in June 2012 in Saudi Arabia. All work with infectious SARS-CoV-2 or MERS-CoV was performed in a Class II Biosafety Cabinet under BSL-3 conditions at Erasmus MC.

Cell lines

Calu-3 cells (a human lung epithelial cell line, ATCC HTB-55) were maintained in Opti-MEM I (1x) + GlutaMAX (Gibco), supplemented with 10% FBS, penicillin (100 IU/mL) and streptomycin (100 IU/mL) in a humidified CO_2 incubator at 37°C. Cells were plated in 96-wells plates (Greiner) in maintenance medium supplemented with Y-27632 apoptosis inhibitor (MedChemExpress). Cells were tested regularly for mycoplasma.

Virus titration

Stock titers of SARS-CoV-2 and MERS-CoV were determined by preparing a 10-fold serial dilution of the stock in OptiMEM I (1x)) + GlutaMAX (Gibco), supplemented with penicillin (100 IU/mL) and streptomycin (100 IU/mL) and adding 100 uL to Calu-3 cells. After 8 hours of incubation at 37°C, cells were fixed in 4% formalin and permeabilized with 70% ethanol with isopropanol. Staining and quantification of positive cells was performed as described under 'Focus Reduction Neutralization Test'.

Enzyme-Linked Immunosorbent Assay (ELISA)

Binding antibodies (IgG) were measured against SARS-CoV-2 full spike (R&D systems; 10853-CV), SARS-CoV-2 S1 (R&D systems; 10609-CV), SARS-CoV-2 S2 (R&D systems; 10640-CV), MERS-CoV full spike (provided by Keith Chapell, University of Queensland, Australia), MERS-CoV S1 (R&D systems; 10737-CV), and MERS-CoV S2 (Sino Biological; 40070-V08B) by an in-house developed ELISA. High binding 96-well ELISA plates (Costar) were coated with 1 ug/mL of the antigen in PBS at 4°C overnight. After coating, plates were blocked for 1 hour at 37°C and incubated with the serum samples in duplicate for 1 hour at 37°C. After incubation, plates were washed and the secondary antibody, Rabbit anti-human IgG-HRP (DAKO) was added. Plates were incubated at 37°C for 1 hour, and developed by using 3,3',5,5'-Tetramethylbenzidine (TMB) (KPL). Signal was measured by an ELISA microtiter plate reader

at an OD of 450 nm. Background signal at an OD of 620 was subtracted. The cutoff was defined as 2*(average OD value pre-pandemic group) + 2*(standard deviation OD value pre-pandemic group). The resulting value was interpolated in 4 replicates of standard curves using an in-house standard for SARS-CoV-2, or the WHO international standard for MERS-CoV, respectively, and the average value in AU/mL or IU/mL was used as cutoff for positivity compared to the pre-pandemic cohort. The lower limit of quantification (LLOQ) was arbitrarily set at 0.15 AU/mL and 0.15 IU/mL, a value that was chosen to be within the range of linearity on the standard curves. Upper Limit of Quantification (ULOQ) was determined per ELISA by plotting the standard curve in PRISM and determining the last point of linearity. Samples that were above the ULOQ, were repeated in a higher dilution to reach the quantifiable (linear) range of the standard curve. All samples that were positive on the in-house MERS-CoV S1 ELISA and all samples that were able to fully neutralize MERS-CoV were tested on a commercial Anti-MERS-CoV IgG S1 ELISA (Euroimmun) according to the manufacturer's protocol using the assay controls supplied by the manufacturer.

Focus Reduction Neutralization Test (FRNT)

Sera were serially diluted threefold and twofold for SARS-CoV-2 and MERS-CoV respectively, starting at a dilution of 1:20 in OptiMEM I (1x)) + GlutaMAX (Gibco), supplemented with penicillin (100 IU/mL) and streptomycin (100 IU/mL). All samples were tested in duplicate. A virus mix containing 400 infectious particles was made based on the 8-hour titration and added to the dilution plate. The mixture of virus and serum was incubated for 1 hour at 37°C. Calu-3 cells were washed and 100 uL of virus-serum mixture was added. After an 8-hour incubation at 37°C, plates were fixed in 4% formalin and permeabilized with 70% ethanol with isopropanol. Fixed and permeabilized plates were washed in PBS. Cells were stained with rabbit anti-SARS-CoV-2 nucleocapsid antibody (Sino Biological) or rabbit anti-MERS-CoV nucleoprotein antibody (GeneTex) dissolved in 0.5% Bovine Serum Albumin (BSA; Sigma-Aldrich) for 2 hours. After removing the primary antibody, secondary antibody goat anti-rabbit antibody (Invitrogen) dissolved in 0.5% BSA was added and incubated in the dark for 2 hours. Next, the nuclei were stained with Hoechst (ThermoFisher) for 30 minutes. After incubation, Hoechst was removed and cells were kept in PBS. The number of virus infected cells was quantified using the Opera Phenix spinning disk confocal HCS system and Harmony software. $FRNT_{50}$ values were calculated using GraphPad PRISM 10 software.

Statistics

Statistical analysis was performed with GraphPad Prism 10 software. Groups were compared by a nonparametric test (Kruskal-Wallis) followed by Dunn's multiple comparisons test. Adjusted P-values are reported. Correlations were determined by the spearman rank-order correlation coefficient.

References

1. Geers D, Aguilar-Bretones M, Zaeck LM, Gill PA, Gonzalez-Lopez C, Handrejk K, et al. Preferential boosting of SARS-CoV-2 Omicron lineage-specific immune responses by monovalent XBB.1.5 vaccination. J Infect. 2026;92:106681. PubMed

2. Gharbharan A, Jordans CCE, GeurtsvanKessel C, den Hollander JG, Karim F, Mollema FPN, et al. Effects of potent neutralizing antibodies from convalescent plasma in patients hospitalized for severe SARS-CoV-2 infection. Nat Commun. 2021;12:3189. PubMed https://doi.org/10.1038/s41467-021-23469-2

Appendix Table 1. Cohort characteristics

Category	Pre-pandemic	Vaccinated	Mild COVID-19	Severe COVID-19
N=	40	40	40	40
Gender	20% Male	30% Male	87.5 % Male	60% Male
Age (median, IQR)	20 (20-21.75)	53 (46.25-56)	46.5 (33.25-56)	64.5 (56-70)
Collection of sera (year)	2015-2018	2023	2020	2020
Exposure to SARS-CoV-2	No	Yes	Yes	Yes
Type of exposure to SARS-CoV-2	-	Vaccination and natural infection	Natural infection	Natural infection
Timing of sera collection (median, IQR)	-	28 (27-28) days after vaccination	59 (41-69.75) days after onset symptoms	1 (0-1) day after ICU admission

Appendix Table 2. Exposures to SARS-CoV-2 in the vaccinated group, 2020-2023

Dosing	Vaccine type
Priming (first vaccine(s) received)	57.5% Ad26.COV2.S, 30% mRNA-1273, 12.5% BNT162b2
Boost bivalent	50% mRNA-1273.214, 15% mRNA-1273.222, 17.5% BNT162b2.BA1, 15% BNT162b2.BA5
Boost XBB.1.5	100%
Breakthrough infection	82.5%

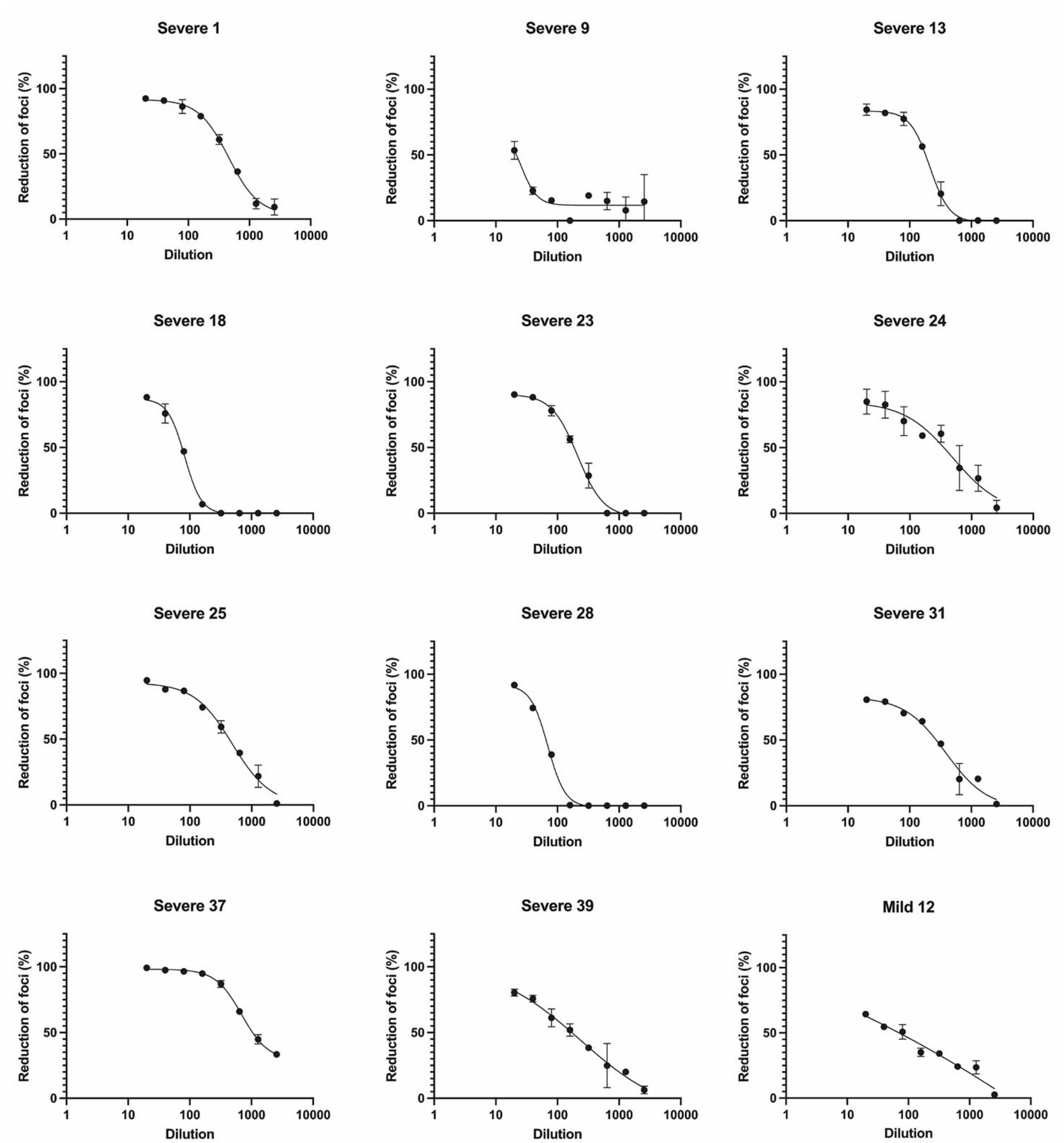

Appendix Figure 1. FRNT S-curves of individuals able to neutralize MERS-CoV, used to determine the FRNT50 titers. Sera from most severe COVID-19 patients able to neutralize MERS-CoV reduce the number of infected foci by >90%. Individuals ICU 9 and MILD 12 do not show full reduction in the lowest dilution, which resulted in a lower titer (26 en 56 respectively).

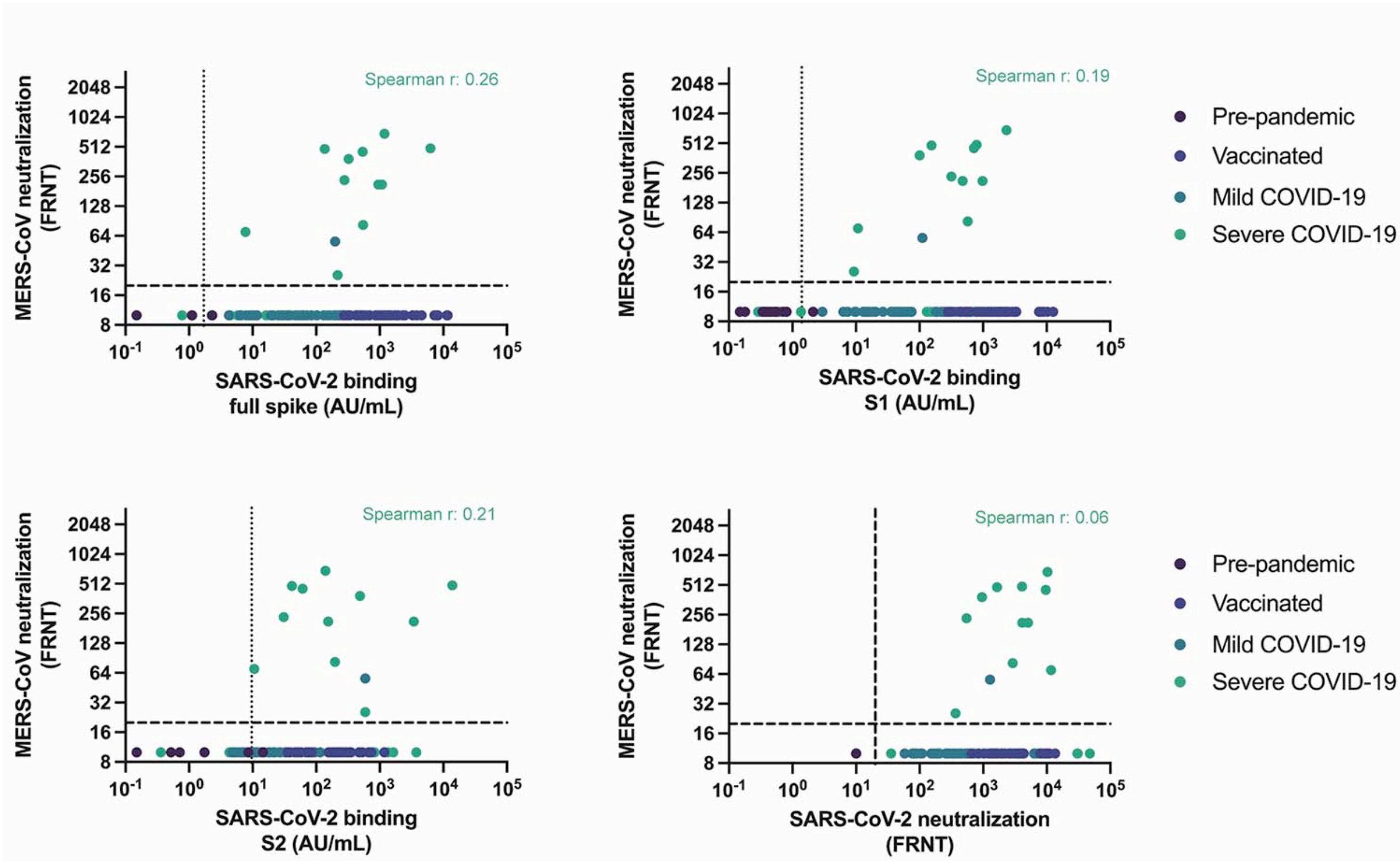

Appendix Figure 2. Correlation plots of MERS-CoV neutralization and SARS-CoV-2-binding IgG antibodies. Sera from all groups was visualized. For individuals with severe COVID-19 (n=40), the correlation was determined by the spearman rank-order correlation coefficient. The spearman r value is indicated in the scatter plot; all correlations were non-significant. Dotted lines indicate the cutoff for positivity based on pre-pandemic samples for in-house ELISAs and dashed lines indicate the lower limit of detection of the FRNT.

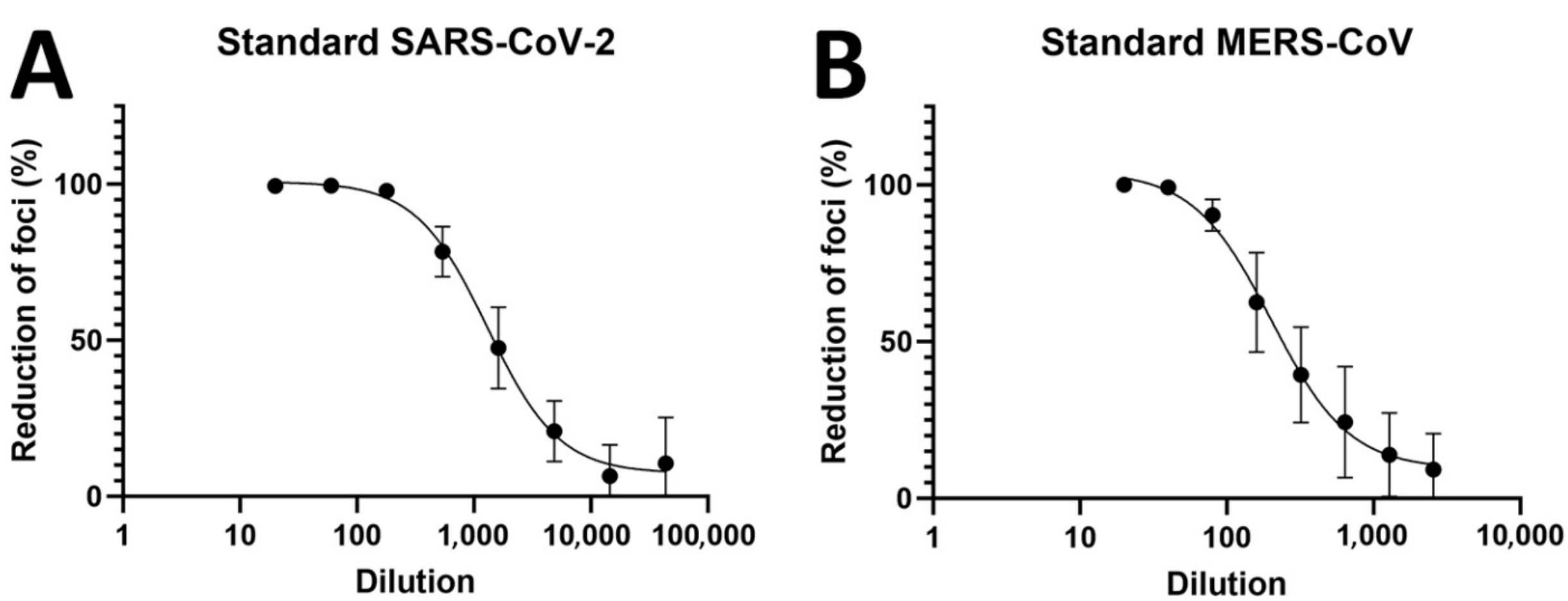

Appendix Figure 3. S-curves of the standards that were included in the FRNT.
